# Biocompatible MIP-202 Zr-MOF tunable sorbent for cost-effective decontamination of anionic and cationic pollutants from waste solutions

**DOI:** 10.1038/s41598-021-86140-2

**Published:** 2021-03-23

**Authors:** Kamal E. Diab, Eslam Salama, Hassan Shokry Hassan, Ahmed Abd El-moneim, Marwa F. Elkady

**Affiliations:** 1 Nanoscience Department, Institute of Basic and Applied Sciences, Egypt-Japan University of Science and Technology, New Borg El-Arab City, Alexandria 21934 Egypt; 2 Environment and Natural Materials Research Institute (ENMRI), City of Scientific Research and Technological Applications (SRTA-City), New Borg El-Arab City, Alexandria 21934 Egypt; 3 Electronic Materials Research Department, Advanced Technology and New Materials Research Institute (ATNMRI), City of Scientific Research and Technological Applications (SRTA-City), New Borg El-Arab City, Alexandria 21934 Egypt; 4 Environmental Engineering Department, Egypt-Japan University of Science and Technology, New Borg El-Arab City, Alexandria 21934 Egypt; 5 Fabrication Technology Research Department, Advanced Technology and New Materials Research Institute (ATNMRI), City of Scientific Research and Technological Applications (SRTA-City), New Borg El-Arab City, Alexandria 21934 Egypt; 6 Chemical and Petrochemical Engineering Department, Egypt-Japan University of Science and Technology (E-JUST), New Borg El-Arab City, Alexandria 21934 Egypt

**Keywords:** Nanoscale materials, Environmental chemistry, Materials science

## Abstract

This reported work aims to fabricate an eco-friendly Zr bio-based MOF and assessment its adsorption efficiency towards the cationic and anionic dye pollutants including methylene blue (MB) and direct red 81 (DR-81), respectively. Also, its adsorption tendency for the highly toxic heavy metal of hexavalent chromium (Cr(VI)) was compared with dyes. The adsorption performance of bio-MOF showed that the maximum monolayer adsorption capacities were recorded as 79.799 mg/g for MB, 36.071 mg/g for DR-81, and 19.012 mg/g for Cr(VI). Meanwhile, the optimum dosage of as-synthesized MIP-202 bio-MOF was 0.5, 1, and 2 g L^−1^ for MB, DR-81, and Cr(VI), respectively. Thermodynamic analysis demonstrated the spontaneous, thermodynamically, and endothermic nature of the decontamination processes onto the fabricated Zr bio-based MOF. The adsorption data were fitted by Langmuir isotherm model compared with Freundlich and Temkin models for all studied water pollutants. Pseudo-second-order kinetic model was a fit model for description of the adsorption kinetics of the different cationic and anionic pollutants onto Zr bio-based MOF. These outcomes indicated that Zr bio-based MOF has potential application for adsorption of different types of industrial water pollutants including cationic and anionic dyes and heavy metals.

## Introduction

Metal–organic frameworks (MOFs), a large class of crystalline and porous hybrid materials, are constructed by linking metal containing units [Secondary Building Units (SBUs)] with organic linkers, using strong bonds (reticular synthesis)^1^. Due to the outstanding properties of MOFs such as high surface areas^2^, tunable pore sizes^3^ and pre or post-modification abilities^1, 4^, MOFs have attracted great interest in modern material science compared with the other nanomaterials^5–7^. MOFs have been studied for the past two decades as promising porous materials for many applications such as catalysis^8^, drug delivery^9^, separation/adsorption^10^ and water purification^11^. Particularly, Zr-carboxylate MOFs which are constructed of zirconium clusters (i.e., Zr_6_ nodes) showed a highly robust and significant performance in various applications^12^. Among these applications, water purification is considered one of the crucial applications due to the straightforward increase in water pollution^7, 13^. As a result, half of the world’s population may live in water-stressed regions by 2025^14^; therefore, the design of excellent and efficient adsorbent MOFs for water purification, combining biocompatibility, excellent stability and cost-effective production is desperately needed for a more clean ecosystem and a sustainable future also, this is still a challenging target at water treatment sector^15^.

In this context, Zr-based MOFs are promising materials for water treatment because of their high porosity that facilitates adsorption and contributes to rapid pollutants removal^11^, including decontamination of chromium from water^16^. The precise tunability of chemical functionality such as amino group inside the 3d ordered pores (e.g., the amino group UiO-66-NH_2_) demonstrates exceptional removal capacity of positive and negative toxic dyes^17^. However, to date, most the vast majority of Zr-MOFs used in water purification are constructed of organic ligands derived from petrochemical sources which are not bio-derived such as 2-amino terephthalic acid, biphenyl-4,4-dicarboxylic acid and trimesic acid in UiO-66-NH_2_^18^, MOF-67^19^, and MOF-808^20^, respectively. Besides, most of Zr-MOFs used in water treatment processes are prepared using toxic organic solvents such as DMF^11^, or acid modulators such as acetic acid or formic acid^21^. So, these MOFs are considered risky and not environmentally benign^22^ and they hinder many important applications requiring eco-friendly materials, such as applications in the biomedicine, food industry and safe water purification^23^. Despite a relatively large number of reports on the removal of organic dyes from water, the interest in probing MOFs utilization for the removal of hexavalent chromium is rapidly growing^24^. We targeted the removal of MB, DR-81, and Cr(VI) due to their profound adverse effects. Not only they are well-known as carcinogen pollutants but also, they are considered as three dangerous pollutants for the water resources^25^.

In this research area, the design of highly efficient biocompatible adsorbent MOFs with no toxicity, excellent moisture stability and scalable eco-friendly preparation method is still a challenging target. Herein, we report for the first time the comparable excellent adsorption performance of a biocompatible Zr-MOF (MIP-202) constructed of aspartic acid as an organic bio-ligand and non-toxic metal ions (Zr(IV)) toward MB, DR-81, and Cr(VI) decontamination from polluted water with high reusability and a very low cost compared to other MOFs.

## Results and discussion

### Characterization of the as-synthesized bio-MOF (MIP-202)

**Figure 1:**
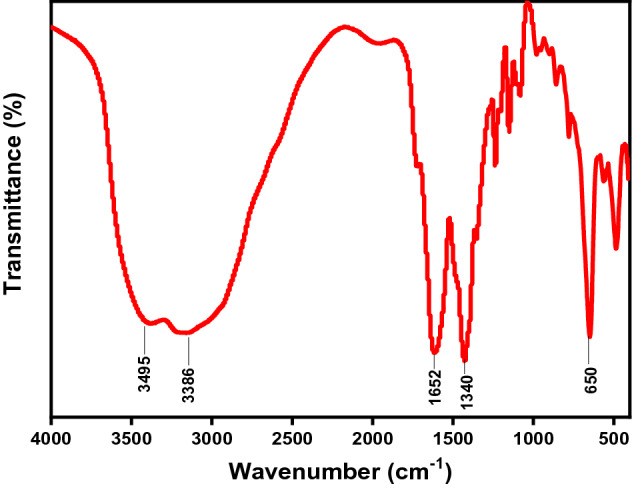
FTIR spectra for the as-synthesized MIP-202 bio-MOF.

FT-IR spectrum of the as-synthesized MIP-202 (Fig. 1) demonstrates the characteristic peaks of bio-MOF included the C–O stretching for the carboxylate groups at 1652 cm^−1^. The C–C stretching was observed at 1568 cm^−1^^26^. The emerging double characteristic peaks at 3495 cm^−1^ and 3386 cm^−1^ are ascribed to the asymmetric and symmetric vibration of the −NH_2_ groups, while the peaks at 1590 cm^−1^ and 1340 cm^−1^ in the lower frequency region corresponding to the −NH_2_ bending vibration and the C−N stretching, respectively^16, 27^. Hence, FT-IR spectrum indicates the presence of the characteristic peaks of as-synthesized MIP-202 bio-MOF.

**Figure 2:**
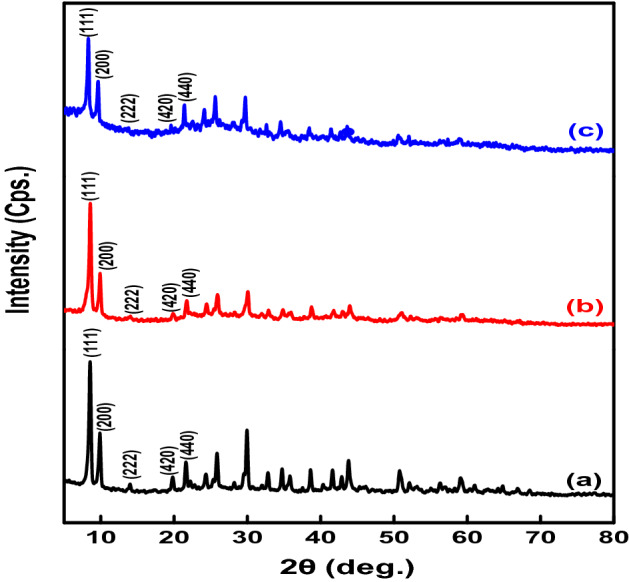
PXRD diffraction patterns of (**a**) as-synthesized MIP-202 bio-MOF, (**b**) as-synthesized MIP-202 bio-MOF soaked in water for 24 h, and (**c**) MOF-801.

The crystalline structure of as-synthesized MOF provided by powder X-ray diffraction (PXRD) was shown in Fig. 2a. The PXRD patterns exhibit distinct peaks at 8.5°, 9.9°,13.9°,19.9°, 21.7°, which can be assigned to the (111), (200), (222), (420) and (440) planes. These characteristic peaks correspond to the most prominent and characteristic diffraction signals of the simulated crystalline MIP-202 bio-MOF structure^28^. XRD data recorded for the as-synthesized MOF matched and showed a good agreement to the XRD data illustrated at Fig. 2c that previously reported for cubic (fcu topology) crystalline MOF-801 (Zr-fumarate)^10, 22^. It is worth mentioning that the crystalline structure of as-synthesized MIP-202 is robust and stable in water. To prove that, the powder was soaked in water for 24 h and there was no change in the crystalline structure as shown in Fig. 2b. As a result, as-synthesized MOF is considered an excellent candidate in water treatment applications and could open the door for future applications in MOFs mixed matrix membranes^27^.

**Figure 3:**
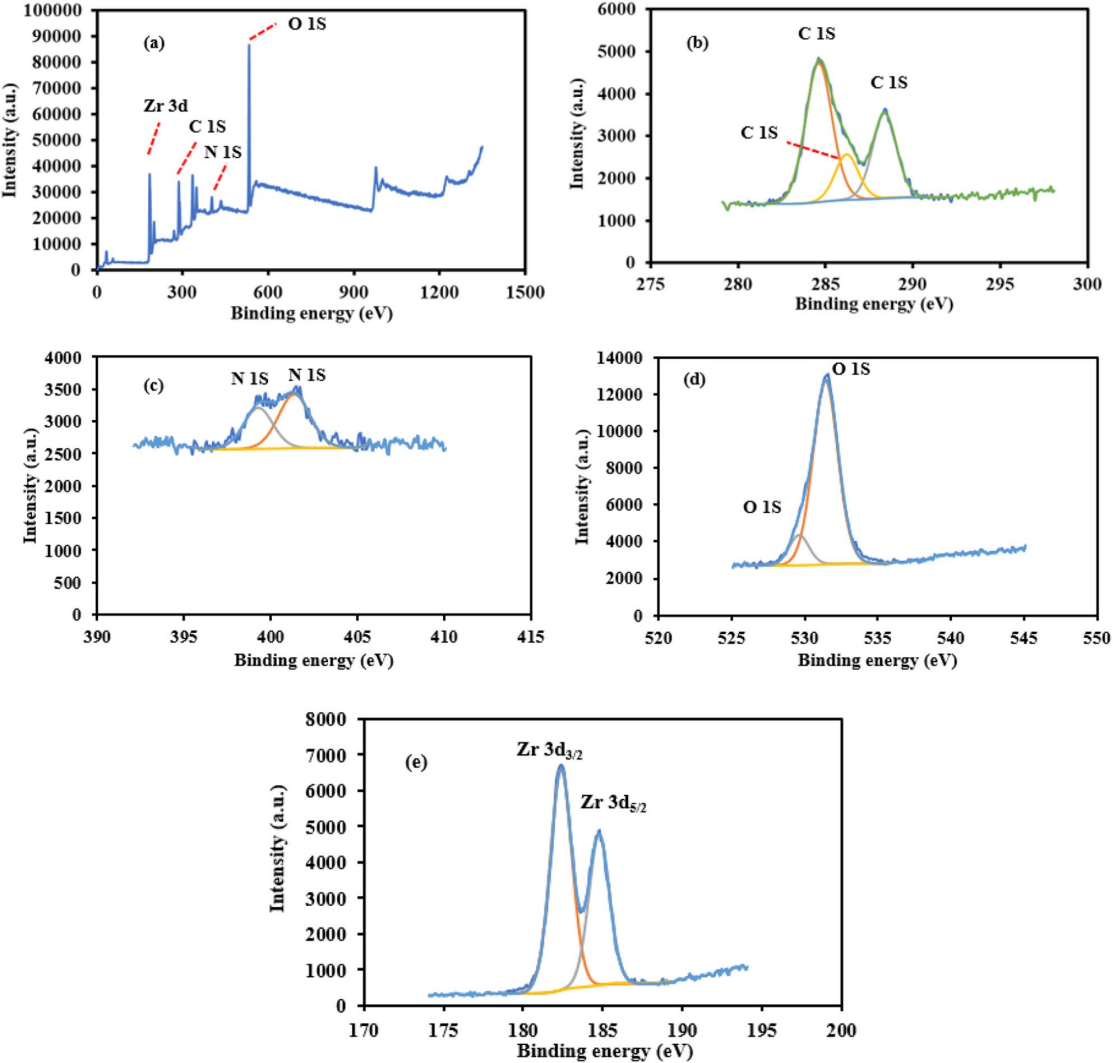
(**a**) XPS spectra of MIP-202 bio-MOF, (**b**) C 1S spectra, (**c**) N 1S spectra, (**d**) O 1S spectra, and (**e**) Zr 3d.

The chemical assembly of the MIP-202 bio-MOF was explored using X-ray photoelectron spectroscopy (XPS) in Fig. 3. The identified N 1s peak at the prepared bio-MOF designated the existence of both –NH_2_ and –NH_2_/NH_3_^+^ (H-bonded with ammonium)^29^. The value of binding energy of the N 1s that ranged to 399.3 eV is characteristic for −NH_2_ groups; however, H-bonded and/or quaternary ammonium represented by the binding energy ranged at 400 eV. The deconvoluted N 1s curves, indicating H-bond interactions within the nano-cages of the material. The O 1s spectrum for the as-synthesized bio-MOF showed a typical BE at 533 eV, which can be addressed to bridging hydroxyl (μ_3_-OH), 531.7 eV ascribed to Zr carboxylate, and 530.2 eV assigned to (μ_3_-O) in Zr–O–Z^30^.

**Figure 4:**
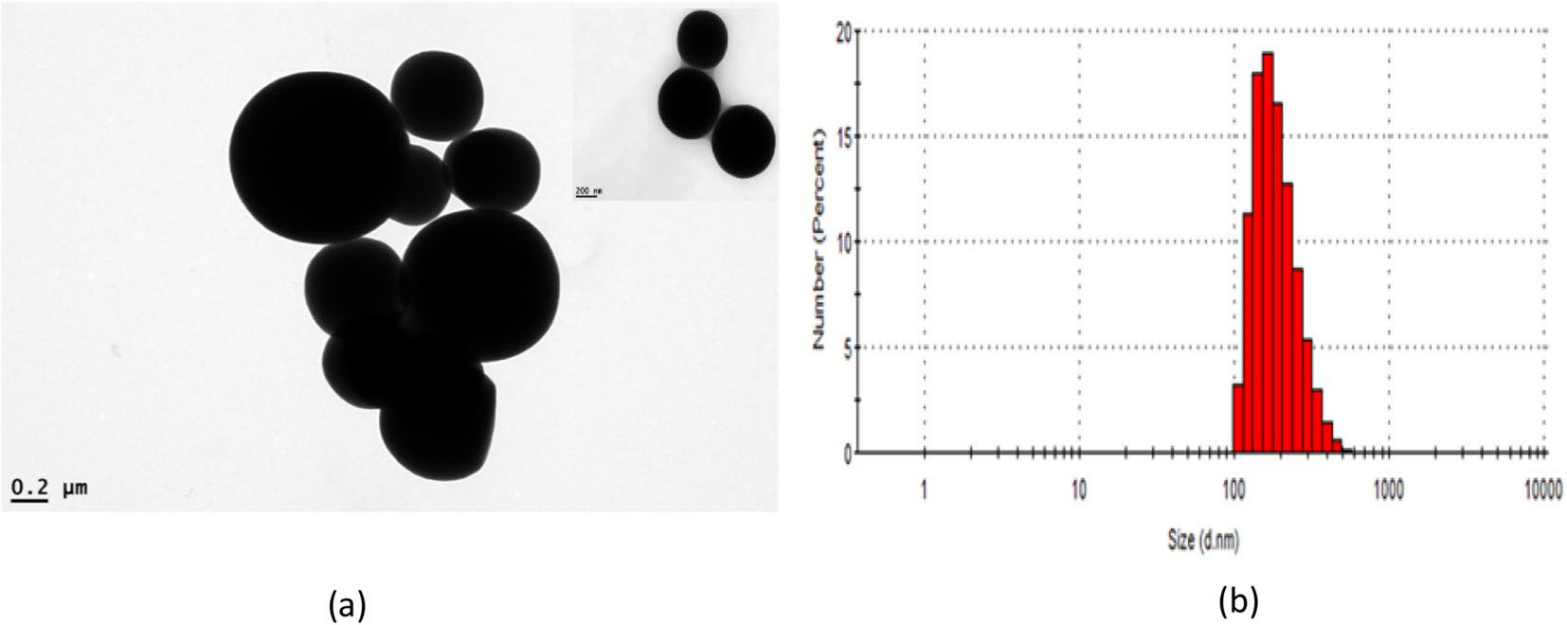
(**a**) TEM images, (**b**) Particle size distribution by number of as-synthesized MOF.

Scanning electron microscopy (SEM) and transmission electron microscopy (TEM) images of the as-synthesized MIP-202 bio-MOF are shown in Figs. S1 and Fig. 4, respectively. The SEM images show the sphere-like morphological structure of the prepared material with average particles size of 356 ± 61 nm. This result was confirmed through the particle size distribution (Fig. 4b) that shows that particle sizes distribution of MIP-202 bio-MOF ranging from 95 to 652 nm with average particles size around 250 nm. This identified nano-size morphology of the prepared bio-MOF with smaller particle sizes and good size uniformity is differ completely compared to the previously reported MIP-202 bio-MOF^22^ that was prepared in micro-scale with a broad particle size distribution. This particle size reduction at as-synthesized MIP-202 bio-MOF could be attributed to the modifications in the preparation conditions. Where, the addition of NaOH that acts as modulator with a NaOH/aspartic acid with a molar ratio of 1:1 in water beside the improvement at both the stirring speed and time under reflux give a high opportunity for the formation of smaller nanoparticles with good production yield compared with the preparation method followed at the literature^27^. The addition of a basic modulator such as NaOH enhanced the dissociation of aspartic acid through the deprotonation of dipolar carboxylic group and thus significantly increased linker solubility in water, which improved the reaction yield and the particle surface area^31^.

Moreover, the elemental analysis of as synthesized MIP-202 bio-MOF using EDS technique was presented in Fig. S2. The results revealed that the material included mainly C, O and Zr with small chlorine content which is still not completely removed due to the high interaction between Chloride ions and + NH_3_ through the formation bio- MOF pores.

The apparent Brunauer–Emmett–Teller (BET) surface area values for as-synthesized bio-MOF calculated from the N_2_ isotherms was shown in Fig. S3. The specific surface area of as-synthesized bio-MOF is approximately 49.5 m^2^ g^−1^. The experimental total pore volume was measured to be 0.053 cm^3^ g^−1^. BET measurements were conducted to prove the permanent porosity of as-synthesized bio-MOF, it is worth mentioning that the isotherm of N_2_ and surface area surpasses the previously reported MIP-202^32^. This could be attributed to modification at the preparation conditions that corresponding to the produced nano-sized bio-MOF beside the high surface area. As it is expected that the good washing of powder beside the assisted sonication after powder production could help in improving material pores^33^. Also, the activation of as-synthesized bio-MOF powder by heating the sample at 100 °C under vacuum for 2 h may enhance the material surface area^10, 34^.

Thermogravimetric analysis (TGA) was conducted in nitrogen to evaluate the thermal stability of as-synthesized bio-MOF. As shown in Fig. S4, various weight-loss degradation steps were observed. The first step weight loss step (∼20%) occurs at temperatures lower than 240 °C and it is attributed to the loss of residual water molecules and atmospheric gases that are trapped into the pores while bio-MOF preparation^13, 32^. This could also be attributed to water adsorbed while synthesis and washing process which cause hydrogen-bonded aggregates and could be reached to 10 H_2_O molecules^13, 22^. The second gradual weight loss step (∼56%), mainly corresponding to the decomposition of the organic framework between 250 and 450 °C, implies the excellent thermal stability of as-synthesized bio-MOF. Comparing the thermal stability of as-synthesized bio-MOF with Zr-based MOFs used in water treatment, it was evident that the as-synthesized MIP-202 bio-MOF showed higher thermal stability than most of the previously reported ones based on di-topic aromatic carboxylic ligands which are not biobased. Also, to the best of our knowledge, this is the first time to report a high efficiency crystalline metal-amino acid biobased porous material, which displays not only excellent stability in the presence of water but also a remarkable resistance to high thermal conditions.

### Application of as-synthesized MIP-202 bio-MOF for environment pollutants’ adsorption

The feasibility of as-synthesized MIP-202 bio-MOF was examined for the adsorption of MB, DR-81, and Cr(VI) from aqueous solutions using batch technique at room temperature^35^.

### Effect of contact time on adsorption processes

**Figure 5:**
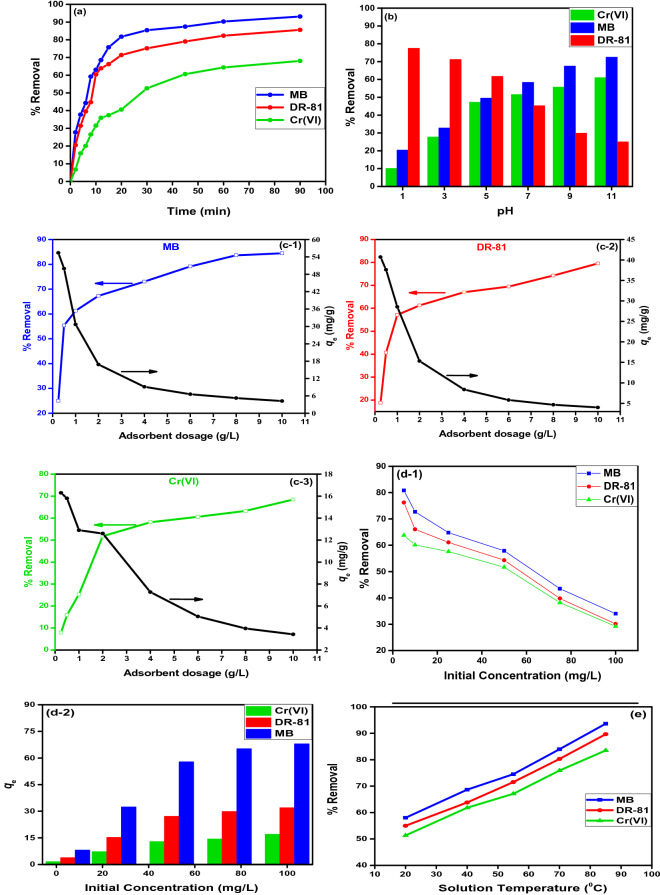
Influence of MB, DR-81, and Cr(VI) removal processing parameters using as-synthesized MIP-202 bio-MOF: (**a**) kinetics of adsorption processes, (**b**) pH, (**c**) sorption dosage, (**d**) initial pollutant concentration, (**e**) solution temperature.

Figure 5a shows the kinetic relationship between MB, DR-81, and Cr(VI) adsorption rate onto as-synthesized MIP-202 bio-MOF at a time interval from 0 up to 90 min. It was illustrated that the removal efficiency of MB, DR-81, and Cr(VI) onto the as-synthesized bio-MOF increased over time till reaching the equilibrium. The enhancement of the adsorption rate in the first stage may be due to the high available surface area and functional groups of the as-synthesized bio-MOF material which binding with the pollutants’ molecules^36^. The equilibrium adsorption time onto as synthetized bio-MOF was determined as 8, 10, and 30 min for MB, DR-81, and Cr(VI), with a maximum removal percentage of 59.19, 60.41, and 52.60%, respectively. After these equilibrium adsorption times, the active sites of the as-synthesized bio-MOF were saturated with MB, DR-81, and Cr(VI) molecules constituting a non-significant adsorption process^35^.

### Effect of initial pH on the adsorption processes

The acidity of waste solution plays an essential role in adsorption processes onto as-synthesized MIP-202 bio-MOF. The pH parameter influences both the ionization degree of pollutants and the surface charge of the adsorbent^37^. As shown in the Fig. S6, the point of zero charge of MIP-202 bio-MOF was recorded at 7.4. Consequently, at any pH below the pzc, the surface of MIP-202 bio-MOF will be positively charged; and above the pzc, the material will be negatively charged. This result was confirmed through zeta potential distribution indicated at Fig. S6. The influence of pH on the adsorption processes was examined at various pH values from 1 to 11. It was observed from Fig. 5b that the acidic conditions were favorable for the DR-81 adsorption. Meanwhile, the basic conditions were favorable for the MB and Cr(VI) adsorption onto as-synthesized MIP-202 bio-MOF. The adsorption of DR-81 was increased up to pH = 5 with maximum adsorption of 61.70% then it was gradually decreased to 24.79% at pH 11. So, the solution pH = 5 was determined as optimum pH for the adsorption of DR-81 onto as-synthesized MIP-202 bio-MOF. In a similar manner, the solution pH = 7 and 9 were selected as optimum pH values for the adsorption of Cr(VI) and MB, respectively. At low pH values ( pH < pzc) the surface of the as-synthesized MIP-202 bio-MOF is positively charged, so there is an electrostatic attraction force between the positive charged surface of as-synthesized MIP-202 bio-MOF and the negatively charged species of DR-81 compared with the repulsion forces for positively charged MB and Cr(VI)^38^. Furthermore, as the solution pH increased (pH > pzc), the density of negative charges increased at the adsorbent surface, which results in adsorption enhancement of positively charged MB and Cr(VI) due to the attraction forces between the negatively hydroxyl and carbonyl groups at as-synthesized MIP-202 bio-MOF and the cationic pollutants^39^.

### Effect of as-synthesized MIP-202 bio-MOF dosage on the adsorption processes

The adsorbent dosage is one of the most influenced parameters in the adsorption processes due to its effects on the adsorbent material capacity^37^. The influence of adsorbent dosage of as-synthesized MIP-202 bio-MOF was tested after 8, 10, 30 min for MB, DR-81, and Cr(VI), respectively. Figure 5c demonstrates that the MB, DR-81, and Cr(VI) removal was enhanced as-synthesized MIP-202 bio-MOF dosage increased from 0.25 g to 10 g/L. On the other hand, the adsorption capacities toward the different water pollutants were decreased with an increasing amount of MIP-202 bio-MOF. The decline at the material adsorption capacity towards the various studied pollutants at high adsorbent dosage may be returned to the unsaturated adsorption residual sites onto the as-synthesized MIP-202 bio-MOF^40^. However, the increment at as-synthesized MIP-202 bio-MOF dosage increases the availability of more and more active sites for pollutant adsorption that increases the percentage removal of water pollutants. These results may be attributed to the relatively high surface area of the as-synthesized MIP-202 bio-MOF which is recorded as 49.5 m^2^ g^−1^^36^. Therefore, the optimum dosages of as-synthesized MIP-202 bio-MOF were selected as 0.5, 1, and 2 g/L for MB, DR-81, and Cr(VI), respectively, which indicated as economical dosages for the adsorption processes.

### Effect of initial concentrations of pollutants on the adsorption processes

The impact of initial concentrations of MB, DR-81, and Cr(VI) was determined in the initial concentration intervals from 5 to 100 mg/L in presence of optimum material dosages, contact time, and pH for each pollutant, separately. Figure 5d investigated that the adsorption capacities ware improved as the initial concentration raised from 5 to 100 mg/L, which completely agrees with the other reported studies^35, 36, 41, 42^. This behavior may be returned to the saturation of the active sites of the as-synthesized MIP-202 bio-MOF as the initial MB, DR-81, and Cr(VI) concentrations increased. From these results, it was clear that the as-synthesized MIP-202 bio-MOF is effectively capable of decontamination of different water pollutants including MB, DR-81, and Cr(VI) from aqueous solutions at different initial concentrations.

### Effect of solution temperature on the adsorption processes

As shown in Fig. 5e, the influence of the temperature on the MB, DR-81, and Cr(VI) removal onto the as-synthesized MIP-202 bio-MOF was studied. It was demonstrated that the MB, DR-81, and Cr(VI) adsorption was raised with increasing the solution temperatures, indicating the favorable adsorption process at high temperatures. This may be due to the improvement in the rate of MB, DR-81, and Cr(VI) diffusions into the pores of the bio-MOF at higher temperatures through the new adsorption sites onto the as-synthesized MIP-202 bio-MOF^41, 43, 44^. These results demonstrate that the MB, DR-81, and Cr(VI) adsorption onto the as-synthesized MIP-202 bio-MOF is an endothermic process^35^.

### Thermodynamics modeling of adsorption processes

To investigate the nature of adsorption processes, different thermodynamic parameters should be determined such as the standard free energy (Δ*G*°), changes in enthalpy (Δ*H*°), and entropy (Δ*S*°). Values of the standard enthalpy and entropy can be determined from Van’t Hoff equation;

1 $$\ln K_{c} = \frac{{\Delta S^{o} }}{R} - \frac{{\Delta H^{o} }}{RT}$$

where *K*_c_ = *F*_e_/(1 − *F*_e_), and *F*_e_ = (*C*_o_ − *C*_e_)/ *C*_o_; is the adsorbed fraction at equilibrium, the universal gas constant (*R*) = 8.314 J/mol.K, while *T* is the temperature of solution in Kelvin. Fig. S5 showed the Van't Hoff relationship for lnK_c_ versus 1000/*T* represented a straight line with an acceptable value of R^2^ in presence of different concentrations of MB, DR-81, and Cr(VI). Δ*H*° and Δ*S*° values can be respectively determined from the slope and the intercept of the plot. Moreover, activation Energy (E_a_) can be calculated from the following equation ^45^:

2 $$E_{{\text{a}}} = \, \Delta H^{0} + RT$$

The calculated thermodynamic parameters of Δ*G*^o^, E_a,_ Δ*H*°, and Δ*S*° for adsorption of MB, DR-81, and Cr(VI) on as-synthesized MIP-202 bio-MOF at constant temperature (358 K) were listed in Table S1. The negative values of Δ*G*° designate the thermodynamically and spontaneous nature of the MB, DR-81, and Cr(VI) decontamination processes onto the as-synthesized MIP-202 bio-MOF^35^. On the contrary, the positive values of enthalpy illustrate the endothermic nature of the adsorption processes. However, the positive values of entropy indicate an enhancement in disorder at liquid/solid interface during the adsorption processes^7, 36^.

### Equilibrium isotherm analysis for adsorption of MB, DR-81, and Cr(VI)

Langmuir, Freundlich, and Temkin models were utilized to investigate the behavior of decontamination processes of MB, DR-81, and Cr(VI) onto as-synthesized MIP-202 bio-MOF. The Langmuir linearized plot of *C*_e_/*q*_e_ against *C*_e_ give a straight-line with a high correlation coefficient (*R*^2^ = 0.990)^41^:

3 $$\frac{{C_{e} }}{{q_{e} }} = \frac{1}{{q_{m} K}} + \frac{{C_{e} }}{{q_{m} }}$$

where *q*_e_ is the amount of MB, DR-81, Cr(VI) adsorbed at equilibrium (mg/g); *C*_e_ is the equilibrium concentration of the adsorbate (mg/L); and *K*_L_ and *q*_m_ are Langmuir constants referred to the adsorption energy (L/mg) and maximum monolayer adsorption capacity (mg/g), respectively. Furthermore, Freundlich linear Eq. (4) was used for analyzing the equilibrium data, by plotting log *q*_e_ versus log *C*_e_^35^.

4 $${\text{Log}}q_{{\text{e}}} = {\text{log}}K_{{\text{F}}} + {1}/n_{{\text{F}}} {\text{log}}C_{{\text{e}}}$$

where *n*_F_ and *K*_F_ are Freundlich constants correlated to the intensity and capacity of adsorption, respectively.

The sorption data of MB, DR-81, Cr(VI) onto the as-synthesized MIP-202 bio-MOF were analyzed with Temkin isotherm model which expressed as following^35^:

5 $$q_{{\text{e}}} = B{\text{ln}}A + B{\text{ln}}C_{{\text{e}}}$$

where *A* is the Temkin isotherm constant (L/g); and *B* = *RT/b* is constant correlates to adsorption heat (J/mol).

Comparing the linearization fitting of the three models at Table S2, it was elucidated that Langmuir model is the most fitted model for representing the decontamination processes of MB, DR-81, and Cr(VI) onto the synthesized bio-MOF. Furthermore, the values of separation factor (*R*_L_) fall between 0 to 1 which indicates the favorable decontamination processes via Langmuir model^35, 46^. Meanwhile, the Freundlich adsorption intensity (*n*_F_) recorded 1.47, 1.63, and 1.62 for adsorption of MB, DR-81, and Cr(VI), respectively, which are greater than the unity, demonstrating that the decontamination processes on the as-synthesized MIP-202 bio-MOF are favorable^46^. On contrary, the low values of Temkin correlation coefficients clarify that the equilibrium data of MB, DR-81, and Cr(VI) adsorption onto the synthesized bio-MOF not fitted with Temkin isothermal model. Therefore, the Langmuir model is the best favorable model for description of the monolayer adsorption of MB, DR-81, and Cr(VI) on the as-synthesized bio-MOF surface^37^.

### Comparison of adsorption capacity for as-synthesized MIP-202 bio-MOF with other adsorbent nanomaterials

The mono-layer adsorption capacities (*q*_m_) of the as-synthesized MIP-202 bio-MOF toward various studied water pollutants were compared with the adsorption capacities of the other similar adsorbent nanomaterials as listed in Table 1. It was evident from the table that the as-synthesized MIP-202 bio-MOF has economic and promising results for adsorption of different water pollutants including MB, DR-81, and Cr(VI) compared with the literature adsorbent nanomaterials.

**Table 1:** Comparison of mono-layer adsorption capacities of MB, DR-81, and Cr(VI) via different adsorbent materials.

Pollutant	Adsorbent material	Optimized conditions	Adsorption capacity (mg/g)	References
MB	As-synthesized MIP-202 MOF	Dose = 0.5 g/L Conc. = 50 mg/L Time = 8 min	79.79	Present study
	Activated carbon	Dose = 0.5 g/L Conc. = 10 mg/L Time = 120 min	53.90	^39^
	MOF based on copper-benzenetricarboxylates (Cu-BTC)	Dose = 0.5 g/L Conc. = 10 mg/L Time = 20 min	15.28	^47^
	UiO-66 MOF	Dose = 0.1 g/L Conc. = 20 mg/L Time = 20 min	13.2	^48^
	Fe-BDC MOF	Dose = 2.5 g/L Conc. = 5 mg/L Time = 300 min	8.65	^49^
DR-81	As-synthesized MIP-202 MOF	Dose = 1 g/L Conc. = 50 mg/L Time = 12 min	36.07	Present study
	Kaolinite	Dose = 4 g/L Conc. = 50 mg/L Time = 120 min	26.55	^50^
	Neem bark	Dose = 0.25 g/L Conc. = 50 mg/L Time = 50 min	8.40	^51^
	Potato peel	Dose = 0.25 g/L Conc. = 50 mg/L Time = 50 min	10.40	^51^
Cr(VI)	As-synthesized MIP-202 MOF	Dose = 2 g/L Conc. = 50 mg/L Time = 30 min	19.01	Present study
	Fe_3_O_4_-GS	Dose = 0.1 g/L Conc. = 3 mg/L Time = 30 min	17.29	^52^
	N-butylacrylate grafted chitosan	Dose = 0.2 g/L Conc. = 50 mg/L Time = 30 min	17.15	^53^
	Azadirachta indica leaves	Dose = 1 g/L Conc. = 75 mg/L Time = 60 min	10.20	^54^

### Kinetic models of MB, DR-81, and Cr(VI) adsorption

In order to investigate the adsorption mechanism of MB, DR-81, and Cr(VI) onto the as-synthesized MIP-202 bio-MOF from aqueous solutions, the pseudo-first order, pseudo-second order, Elovich, and intraparticle diffusion kinetic models were applied. The Lagergren first order equation can be expressed as following^55^:

6 $${\text{ln }}\left( {q_{{\text{e}}} - q_{{\text{t}}} } \right) = {\text{ln}}q_{{\text{e}}} - {\text{k}}_{{1}} t$$

where *q*_e_ and *q*_t_ are the amounts of MB, DR-81, and Cr(VI) adsorbed ions (mg/g) at equilibrium and at time *t* (min), respectively. *k*_1_ (min) is the rate constant of the first-order reaction. Moreover, the decontamination kinetic data were analyzed via the pseudo-second order kinetic model which can be represented as following^36^:

7 $$t/q_{{\text{t}}} = \left( {{1}/k_{{2}} q^{{2}} } \right) + t/q$$

where *k*_2_ is the constant of the second-order rate (g/mg·min). Moreover, Elovich equation was considered for the adsorption of various water pollutants by^35^:

8 $$q_{{\text{t}}} = \alpha + \beta {\text{ln}}t$$

where *α* refers to the initial adsorption rate (mg/g·min) and *β* is correlated to the degree of the surface coverage and activation energy of adsorption (g/mg). *α* and *β* can be respectively determined from the slope and intercept of the linear plot of *q*_t_ against ln *t*. Finally, the possibility of the intraparticle diffusion influencing the adsorption processes for the different studied pollutants was explored by Weber and Morris equation ^56^;

9 $$q_{{\text{t}}} = k_{{\text{i}}} {\text{t}}^{{{1}/{2}}} + C$$

where *k*_i_ is the constant of the intraparticle diffusion rate. Values of C provide prediction about the thickness of the boundary layer. If intraparticle diffusion occurs, then qt versus t1/2 will be linear and if the plot passes through the origin, then the rate limiting process is only due to the intraparticle diffusion. Otherwise, some other mechanism along with intraparticle diffusion is also involved.

The comparable investigation for the correlation coefficient values for linear plotting of the four studied kinetic models for adsorption MB, DR-81, and Cr(VI) ions onto the as-synthesized MIP-202 bio-MOF from aqueous solutions was tabulated at Table S3. It was indicated that the linearity of plotting *t*/*q*_t_ versus time offer high correlation coefficient values for the different studied water pollutants (*R*^2^ = 0.999, 0.999, and 0.997) demonstrates that the adsorption processes of MB, DR-81, and Cr(VI) onto as-synthesized MIP-202 bio-MOF follow the second order rate kinetic model. Furthermore, as evident from the table, the calculated values of *q*_e_ for pseudo-second-order are very close to the experimental values. These results confirm that the MB, DR-81, and Cr(VI) adsorption processes onto as-synthesized MIP-202 bio-MOF were mainly controlled via pseudo-second kinetic model for the different studied water pollutants^57^.

### Adsorption mechanism of MB, DR-81, and Cr(VI) onto synthesized MIP-202 bio-MOF

**Figure 6:**
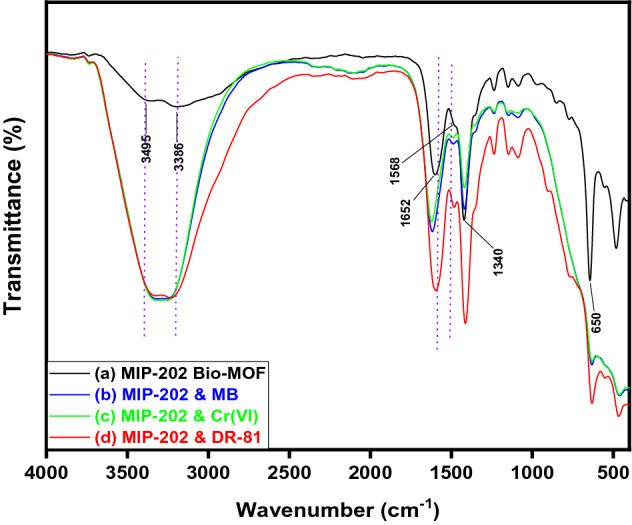
FTIR spectra for (**a**) MIP-202 bio-MOF, (**b**) MIP-202 & MB, (**c**) MIP-202 & Cr(VI), (**d**) MIP-202 & DR-81.

The FTIR spectra before and after adsorption of MB, DR-81 and Cr(VI) were compared as shown in the Fig. 6. After MB, DR-81 and Cr(VI) adsorption, the FTIR spectrum of the MIP-202 bio-MOF presented characteristic changes due to the adsorption of MB, DR-81 and Cr(VI) onto MIP-202 bio-MOF. Comparing the FTIR before and after the adsorption process, it was indicated that the peak representing the C–O stretching for the carboxylate groups of MIP-202 bio-MOF at 1652 cm^−1^ was shifted and the C–C stretching at 1568 cm^−1^ was diminished. This might be due to the chemical formation of bonds between C–C of MIP-202 bio-MOF and electrophilic N^+^ function groups at MB and DR-81 and/or positive Cr ions. Moreover, the peak of Zr–O was shifted to higher wavenumbers confirming the chemical bonds between Zr–O and cationic function groups at dyes and/or Cr ions^58^. Furthermore, the double characteristic peaks at 3495 cm^−1^ and 3386 cm^−1^ of asymmetric and symmetric vibration were shifted confirming the interaction between –NH_2_ group and positively characteristic groups of pollutants16. These outcomes demonstrate that the adsorption mechanism may be controlled by chemical interaction between the synthesized MIP-202 bio-MOF and pollutants.

### Reusability studies of as-synthesized MIP-202 bio-MOF

**Figure 7:**
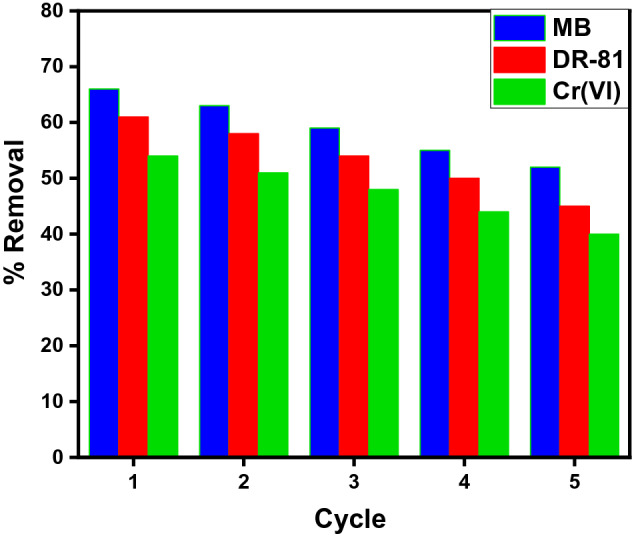
Recyclability test for adsorption of MB, DR-81, and Cr(VI) onto as-synthesized MIP-202 MOF.

The reusability study of the adsorbent materials is one of the most important factors, where it influences the overall cost of the real applications^7^. Accordingly, to evaluate the regeneration processes of as-synthesized MIP-202 bio-MOF, it was reused in the batch experiments for the adsorption of MB, DR-81, and Cr(VI). The adsorption–desorption cycles were repeated five times. According to the obtained data, the as-synthesized MIP-202 MOF proved the ability to be reused several times with high removal efficiency for MB, DR-81, and Cr(VI) as shown in Fig. 7.

### Cost of as-synthesized MIP-202 bio-MOF

Cost is considered an important factor when it comes to evaluating the potential of an adsorbent for practical and manufacturing use especially since this bio-MOF is an eco-friendly one compared to the majority of other MOFs which is not based on bio-based ligand. The cost of MOFs mainly depends on the cost of organic ligands. For instance, some MOFs which are used as a good adsorbent like Mg-MOF-74 costs (2836 $/kg) despite, it's not eco-friendly^58^. On the other hand, as-synthesized MIP-202 could barely cost (36 $/kg) with excellent performance as adsorbent which makes it a promising nanomaterial for manufacturing and practical applications. All price data Table S4 were provided from J&K Scientific Co., Ltd. official website^59^.

## Conclusion

In conclusion, we report herein for the first time an efficient adsorbent porous zirconium aspartate metal–organic framework (MOF) comprises non-toxic metal ions-Zr(IV) and a biocompatible, renewable and cheap linker, aspartic acid. As-synthesized MIP-202 bio-MOF was characterized using FT-IR, PXRD, XPS, SEM, TEM, EDX, BET, and TGA. The as-synthesized MIP-202 bio-MOF is nontoxic and environmentally-benign. Furthermore, the adsorption behavior of MB, DR-81, and Cr(VI) was well fitted with Langmuir model that demonstrated the monolayer adsorption onto as-synthesized MIP-202 bio-MOF. The best kinetic model for MB, DR-81, and Cr(VI) adsorption was pseudo-second-order model. The maximum adsorption capacities on as-synthesized MIP-202 bio-MOF were 79.799, 36.071, and 19.012 mg/g for MB, DR-81, and for Cr(VI), respectively. The as-synthesized MIP-202 bio-MOF is an effective promising adsorbent in the adsorption of MB, DR-81, and Cr(VI) from aqueous solutions with high stability and recyclability for multiple cycles, as well as an easily regenerable form of the sorbent.

## Materials and methods

### Materials

Zirconium tetrachloride (ZrCl_4_, 99.5%) and L-aspartic acid (LA, 99.0%) were obtained from Strem Chemicals Inc. and Sigma Aldrich Co., Ltd, respectively. Ethanol (EtOH, HPLC) was purchased from Fisher Scientific. DR-81 (50% Dye content) with molecular weight 675.60 g/mol and methylene blue (MW: 319.85 g/mol) were purchased from sigma-Aldrich (USA). All chemicals obtained were used without further purification. Pyrex round-bottom flask and reflux were used for the synthesis of bio-MOF material. Pyrex screw-capped media storage jars were used for bio-MOF storage.

### Synthesis of zirconium-L-aspartic amino acid (MIP-202)powdered material

In a 25 mL screw-capped jar, 0.7 g (5.26 mmol) of L-aspartic acid was dispersed in 5 ml of deionized water as a solvent. The dispersed powder of L-aspartic acid was then sonicated in an isothermal oven at 40 °C for 10 min to produce a well-dispersed solution of the organic ligand. In another 25 ml screwcapped jar, 0.57 g (2.465 mmol) of ZrCl_4_ was completely dissolved in 5 ml of deionized water under continuous stirring for 5 min. Afterward, the solution of ZrCl_4_ was added dropwised to the L-aspartic solution under continuous stirring at 200 rpm. Subsequently, the mixture was transferred into a 25 mL round-bottom flask and heated at 373 K under reflux and stirring for 12 h until production of a white precipitate of as-prepared MIP-202. After cooling down to room temperature, the precipitate was collected by centrifugation at 7000 rpm for 10 min, washed three times with 100 mL deionized water and ethanol for three days, and subsequently dried in air. The air-dried MOF sample was transferred to a vacuum chamber. The chamber was firstly evacuated at 25 °C for 30 min, then the prepared sample was heated in vacuum at 70 ºC for 12 h, yielding activated MIP-202 as a white powder material.

### Characterization of as-synthesized bio-MOF(MIP-202)

The chemical structure of as prepared MIP202 was recorded using infrared absorption spectra using FTIR (Thermo-Scientific Nicolet, USA). X-ray spectrum of the bio-MOF was detected using Shimadzu XRD-6100 with Cu–Kα radiation (λ = 1.54 Å) to determine the crystalline structure of the material. To assess the chemical states of the prepared MOF, X-ray photoelectron spectroscopy (XPS, Thermo Fisher Scientific, USA) analysis with X-ray Al k_α_ radiation was used. Photoluminescence spectra were recorded using Agilent Cary Eclipse Fluorescence Spectrophotometer. The morphological structure of the as-synthesized bio-MOF was investigated using scanning electron microscopy (SEM, JEOL JSM-6010LV). Images with high resolution were obtained using transmission electron microscopy (TEM, JEOL JEM-2100F) equipped with energy dispersive X-ray (EDX) spectroscopy to specify the chemical composition of the as-synthesized MOF. The surface area and the pore size distribution of the prepared bio-MOF were determined using Belsorp-max automated apparatus through degassing the material at 200 °C for 5 h before the measurements. The thermal stability of the as-synthesized MOF was evaluated using thermo-gravimetric analysis (TGA-50, Shimadzu). The temperature was increased from 25 to 1000 °C under nitrogen and the sample weight losses were screened over the studied temperature range. The sample heating rate and gas flow rate were 10 °C/min and 40 mL/min, respectively.

### Batch adsorption for water purification

The distinguishing adsorption performance of the as-synthesized MIP-202 bio-MOF was highlighted using various water pollutants including dyes and heavy metals. Comparable investigation of MIP-202 bio-MOF adsorption capacities toward anionic and cationic pollutants was performed using batch technique. Where, 50 mg of bio-MOF was mixed with 25 mL from specific pollutants at various initial concentrations at 20 °C using a shaking incubator. The impact of various processing parameters on the adsorption performance of the as-synthesized MIP-202 including contact time (0–90 min), pH (1–11), adsorbent dosage (0.5–10 g/L), initial pollutant concentration (5–100 mg/L), and reaction temperature (20–85 °C) were monitored. The reliability of the resulted data was confirmed by applying all adsorption experiments in triplicate and the mean values were utilized in data analysis processes. After finishing the water treatment process, the adsorbent material was separated by centrifugation to determine the final pollutant concentration through the colorimetry method using UV spectrophotometer at 665, 465, and 365 nm for MB, DR-81, and Cr (VI) respectively. The pollutant removal percentage by as-synthesized MIP-202 bio-MOF was calculated from Eq. (10)^13,36^:

10 $${\text{Removal}}\% = \left( {\left( {C_{{\text{o}}} - C_{{\text{e}}} } \right)/C_{{\text{o}}} } \right)*{1}00$$

where *C*_o_ is the initial pollutant concentration (mg/L); and *C*_e_ is the pollutant concentration at equilibrium in aqueous solution (mg/L). The pollutant adsorption capacity (mg/g) was calculated from the following equation ^35^:

11 $$q_{{\text{e}}} = V\left( {C_{{\text{o}}} - C_{{\text{e}}} } \right)/m$$

where *q*_e_ is the adsorption capacity of pollutant (mg/g); *V* is the solution volume (L); and *m* is the mass of as-synthesized MIP-202 bio-MOF (g).

### Equilibrium, kinetics and thermodynamics adsorption behavior of bio-MOF

In order to investigate the nature of adsorption processes onto the prepared bio-MOF, thermodynamic parameters of the adsorption process toward the different studied water pollutants were evaluated. Moreover, the equilibrium behavior of the adsorption processes onto as-synthesized MIP-202 bio-MOF was tested using Langmiur, Frendlich and Temkin isothermal equations. Adsorption kinetics were evaluated using pseduo-first-order, pseduo-second-order, Elovich and intraparticle kinetic models.

### Reusability of as-synthesized MIP-2020 bio-MOF

In order to improve the economic feasibility of the water treatment process using the prepared bio-MOF, the utilized MIP-202 bio-MOF material at the adsorption process was regenerated through three times washing with deionized water followed by ethanol then dried in air for further reuse.

## Supplementary Information

Supplementary information.
