# Supplementary material for: Biocompatible MIP-202 Zr-MOF tunable sorbent for cost-effective decontamination of anionic and cationic pollutants from waste solutions
Source: Sci Rep. 2021 Mar 23;11:6619. doi: 10.1038/s41598-021-86140-2 (PMC7987968; doi:10.1038/s41598-021-86140-2)
Supplement: Supplementary file 1 — Supplementary information. [file 41598_2021_86140_MOESM1_ESM.docx]

**Supplementary Materials for**

**Biocompatible MIP-202 Zr-MOF tunable sorbent for cost-effective decontamination of anionic and cationic pollutants from waste solutions**

Kamal E. Diab,^1^ Eslam Salama,^2^* Hassan Shokry Hassan,^3,4^* Ahmed Abd El-moneim,^1^ Marwa F. Elkady^5,6*^

*Corresponding author. Email: [Hassan.shokry@ejust.edu.eg](mailto:Hassan.shokry@ejust.edu.eg) (H.S.); [eslam.salama@ejust.edu.eg](mailto:eslam.salama@ejust.edu.eg) (E.S.); [marwa.elkady@ejust.edu.eg](mailto:marwa.elkady@ejust.edu.eg) (M.E.)

**This PDF file includes:**

Figures S1 to S5

Tables S1 to S4


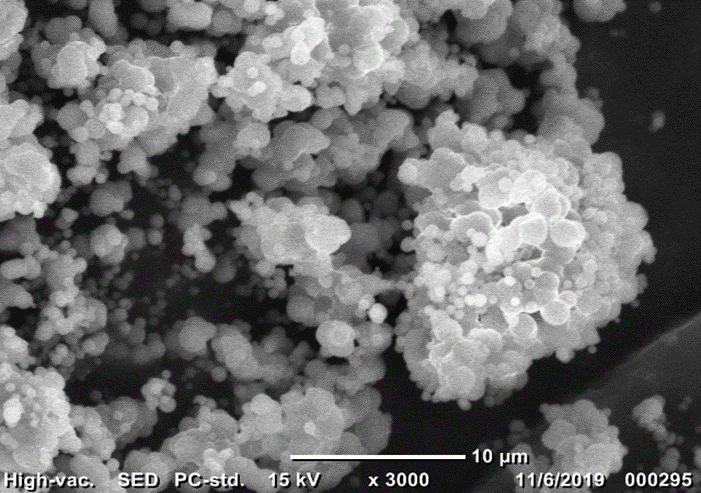

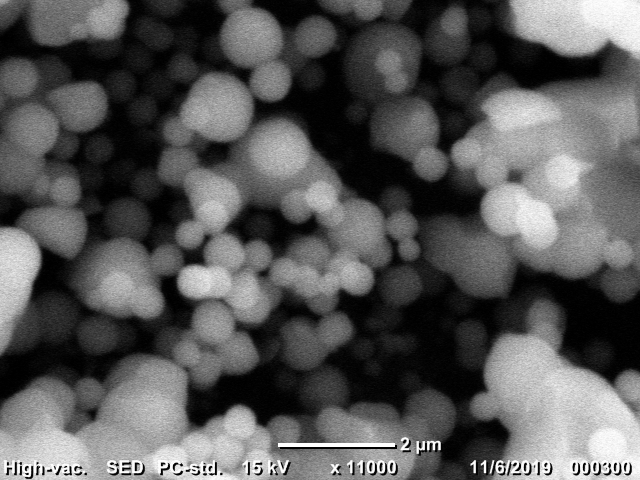


**Figure S1.** SEM images of as-synthesized MOF at different magnifications.


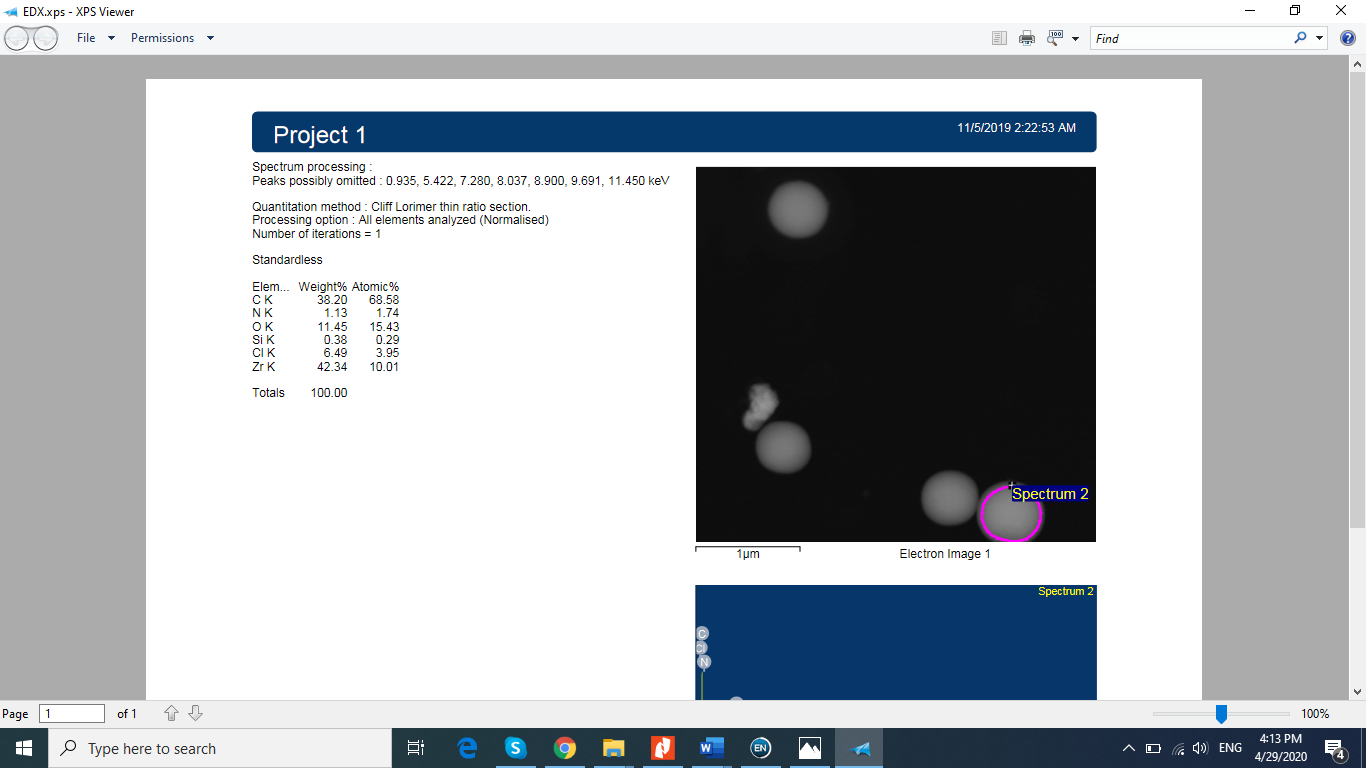


**Figure S2.** EDX analysis of the as-synthesized bio-MOF.

**Figure S3.** N_2_ adsorption−desorption isotherms at 77 K for calculation BET surface areas of as-synthesized bio-MOF.

**Figure S4**. TGA curve of as-synthesized bio-MOF in air at a heating rate of 10 °C/min under temperature ramp mode.

**Figure S5.** Van’t Hoff plot of ln K_c_ against 1000/*T* for MB, DR-81, and Cr(VI) adsorption onto as-synthesized MIP-202 bio-MOF.


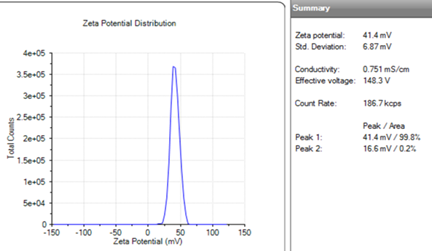


Figure S6. Zeta potential measurements for MIP-202 bio-MOF at different pH values.

| **Pollutant** | **Δ*G*^o^ (kJ·mol^−1^)** | ***E*a (kJ·mol^−1^)** | **Δ*H*^o^ (kJ·mol^−1^)** | **Δ*S*^o^**  **(kJ·mol^−1^)** |
| --- | --- | --- | --- | --- |
| **MB** | -10.07 | 32.61 | 29.63 | 107.68 |
| **DR-81** | -6.43 | 28.23 | 25.25 | 86.34 |
| **Cr(VI)** | -2.76 | 23.41 | 20.43 | 63.71 |

**Table S1.** Thermodynamic parameters for MB, DR-81, and Cr(VI) adsorption onto as-synthesized MIP-202 bio-MOF at constant solution temperature of 358 K.

| **Isotherm parameters** | | **MB** | **DR-81** | **Cr(VI)** |
| --- | --- | --- | --- | --- |
| **Langmuir parameters** | *q*_m_ (mg/g) | 79.799 | 36.071 | 19.012 |
|  | *K*_L_ (L/mg) | 0.096 | 0.087 | 0.056 |
|  | *R*^2^ | 0.997 | 0.994 | 0.990 |
| **Freundlich parameters** | *K*_F_ (mg/g) | 0.534 | 0.534 | 0.533 |
|  | *n*_F_ | 1.476 | 1.631 | 1.625 |
|  | *R*^2^ | 0.886 | 0.942 | 0.970 |
| **Temkin parameters** | *A* (L/g) | 1.280 | 1.066 | 0.692 |
|  | *B* (J/mo) | 15.778 | 7.404 | 4.001 |
|  | *R*^2^ | 0.881 | 0.876 | 0.882 |

**Table S2**. Isotherm parameters of Langmuir, Freundlich and Temkin models for the adsorption of MB, DR-81, and Cr(VI) onto as-synthesized MIP-202 bio-MOF.

| **Kinetic model** | **Parameters** | **MB** | **DR-81** | **Cr(VI)** |
| --- | --- | --- | --- | --- |
| **Pseudo-first order** | *q*_exp_.(mg/g) | 14.798 | 15.101 | 13.151 |
|  | *q*_theor_ (mg/g) | 24.773 | 22.997 | 20.712 |
|  | *K*_1_ (min−1) | 0.187 | 0.157 | 0.077 |
|  | *R*_2_ | 0.965 | 0.972 | 0.981 |
| **Pseudo-second order** | *q*_exp._(mg/g) | 14.798 | 15.101 | 13.151 |
|  | *q*_theor_ (mg/g) | 13.384 | 13.566 | 12.332 |
|  | *K*_2_ (g/mg·min) | 0.158 | 0.132 | 0.052 |
|  | *R*^2^ | 0.999 | 0.999 | 0.997 |
| **Elovich kinetic model** | *α* (mg/g·min) | 4.453 | 2.847 | -42.049 |
|  | *β* (g/mg) | 4.657 | 4.525 | 4.331 |
|  | *R*^2^ | 0.966 | 0.967 | 0.994 |
| **Intraparticle diffusion kinetic model** | *C*_1_ (mg/g·min) | 1.306 | -0.897 | -2.819 |
|  | *C*_2_ (mg/g·min) | 13.239 | 12.843 | 3.114 |
|  | *k*_i_ (g/mg) | 1.941 | 1.893 | 1.931 |
|  | *R*^2^ | 0.877 | 0.882 | 0.966 |

**Table S3**. Pseudo-first order, pseudo-second order, Elovich and Intraparticle diffusion kinetic parameters for MB, DR-81, and Cr(VI) removal onto as-synthesized MIP-202 bio-MOF.

| **MOFs** | **Ligand** | **Price^a^**  **(per/kg)** | **Purity** |
| --- | --- | --- | --- |
| **CoIPA** | isophthalic acid | $105 | 99% |
| **Cu-BTC** | 1,3,5-benzenetricarboxylic acid | $121 | 99% |
| **NJU-Bai35** | isonicotinic acid | $161 | 99% |
| **SIFSIX-3-Zn** | Pyrazine | $510 | 99% |
| **[Cd_2_L(H_2_O)]_2_·5H_2_O** | 4,4′(hexafluoroisopropylidene)diphthalic anhydride | $1975 | 99% |
| **Mg-MOF-74** | 2,5- dihydroxyterephthalic acid | $2836 | 98% |
| **Qc-5-Cu-sql-β** | quinoline-5-carboxylic acid | $45537 | 97% |
| **SIFSIX-14-Cu-i** | ammonium hexafluorosilicate  4,4′-azopyridine | $311  $7744 | 98%  98% |
| **ZU-66** | 4,4′-bipyridylacetylene | $386902 | 95% |
| **NOTT-300** | biphenyl-3,3′,5,5′- tetracarboxylic acid | $561396 | 98% |
| **MIP-202** | L-aspartic acid | $36 | 98% |

**Table S4**. The ligand’s price for some reported MOFs with excellent performance.
